# Detection and Heritability of Genes Conferring Resistance to Potato Pests and Viruses in Hybrid Potato Progeny (*Solanum tuberosum* L.)

**DOI:** 10.3390/plants15162469

**Published:** 2026-08-14

**Authors:** Irina V. Kim, Olga A. Sobko, Petr V. Fisenko, Egor M. Shchelkanov, Nadezhda N. Kakareka, Mikhail Yu. Shchelkanov, Aleksei G. Klykov

**Affiliations:** 1 Federal Scientific Center of Agricultural Biotechnology of the Far East Named After A.K. Chaiki, 30 Volozhenina st., Timiryazevsky stl., 692539 Ussuriysk, Russia; o.eyvazova@gmail.com (O.A.S.); phisenko@bk.ru (P.V.F.); alex.klykov@mail.ru (A.G.K.); 2 Faculty of Natural Sciences, State University of Education, 10A/2 Radio st., 105005 Moscow, Russia; egorshchelkanov@mail.ru; 3 Institute of Epidemiology and Microbiology Named After G.P. Somov, 1 Selskaya st., 690087 Vladivostok, Russia; kakareka@biosoil.ru (N.N.K.); adorob@mail.ru (M.Y.S.)

**Keywords:** *Solanum tuberosum* L., cultivar, hybrid, viruses, markers, resistance genes

## Abstract

Potato viral infections are among the main factors contributing to reduced quality of planting material and decreased tuber productivity. Currently, no reliable chemical control methods are available for plant viral diseases. Therefore, the development of potato cultivars carrying virus resistance genes remains one of the most effective and comprehensive approaches to this problem. In this study, 29 Russian and foreign potato cultivars, as well as 31 Far Eastern potato hybrids were evaluated. Resistance genes were identified using PCR analysis. The following cultivars carrying target resistance genes were used as positive controls for method calibration: Meteor (*Rysto*, *Rx1*, *Sen1*, *Gpa2*, *H1*), Vektor (*Rx1*, *Gpa2*), Yubilyar (*Gpa2*), and Zhukovsky ranniy (*Gpa2*, *Rx1*). Method calibration enabled determination of optimal magnesium chloride concentrations: 2.0 mM for *Gpa2* and 2.5 mM for *Rx1.* Genotyping of 29 potato cultivars identified several highly resistant accessions, including Yubilyar, Zhukovsky ranniy, Bellarosa, Sante, Smak, Red Scarlett, and Laperla. These cultivars combined resistance to Potato virus X (*Rx1*) with complex resistance to two nematode species (*Gpa2*, *H1*). In the studied population, high frequencies of the *Gpa2* (82.8%) gene and the *H1* (65.5–69.0%) gene group were observed. Statistical analysis provided strong evidence for tight genetic linkage between the *Rx1* and the *Gpa2* loci on chromosome 12. The association was highly significant (*p* < 0.001). Analysis of 31 potato hybrids revealed 14 multi-marker genotypes with high breeding potential. A stable combination of five target resistance markers was consistently detected in their genomes. A dominant hybrid family derived from the Yantar × Smak cross was identified, represented by five related lines. For the first time, a precise heritability coefficient was calculated for the STS marker of the *Rx1* gene in a Far Eastern hybrid population. The estimate reached *h*^2^ = 0.835 at *p* = 0.01. This value significantly exceeded the critical threshold for breeding reliability (*h*^2^ > 0.7), indicating largely additive genetic control of the trait. These results support targeted selection of parental combinations for breeding programs aimed at improving virus and nematode resistance in potato.

## 1. Introduction

Potato, *Solanum tuberosum* L., is affected by numerous viral pathogens. Nine of them constitute a major economic concern, as they can cause substantial yield losses and markedly reduce tuber quality. Viral and viroid infections are among the leading factors limiting potato production. To date, more than 30 viruses and one viroid species have been reported to infect potato worldwide [1]. The most common and economically important potato viruses include Potato leafroll virus (PLRV, *Polerovirus*, Sobelivirales: Solemoviridae), Potato virus Y (PVY, *Potyvirus yituberosi*, Patatavirales: Potyviridae), Potato virus X (PVX, *Potexvirus ecspotati*, Tymovirales: Alphaflexiviridae), Potato virus S (PVS, *Carlavirus sigmasolani*, Tymovirales: Betaflexiviridae), and Potato virus M (PVM, *Carlavirus misolani*, Tymovirales: Betaflexiviridae) [2,3].

The prevalence of viral diseases in agricultural ecosystems continues to increase. The Russian Far East is no exception, and potato viral infections have been reported throughout most of the region [4,5]. Viral diseases, particularly PVY, can reduce potato yield twofold to threefold and negatively affect tuber quality [6,7,8,9]. The high pathogenicity, worldwide distribution, and broad genetic diversity of isolates make PVY one of the most significant viral pathogens of cultivated plants [10]. PVX is also widespread in potato-growing regions. In many cases, PVX infection remains asymptomatic, although mild leaf spotting may occasionally be observed. Nevertheless, this virus can significantly reduce tuber yield, with field infection causing average losses of 15–30% [11,12]. Despite the relatively mild symptoms observed in single infections, PVX pathogenicity becomes much more pronounced in mixed infections involving unrelated viruses, typically *Potyvirus* spp. Such synergism increases accumulation of each virus in plant tissues and promotes the development of severe disease symptoms, ultimately leading to substantial yield losses [13].

H. Barker and M.F.B. Dale [14] identified seven principal types of resistance to potato viruses: (1) resistance to infection, also known as horizontal or field resistance; (2) resistance to virus accumulation; (3) resistance to virus movement within the plant; (4) mature plant resistance; (5) tolerance; (6) resistance to virus vectors; (7) hypersensitive response, and extreme resistance. Resistance to infection is characterized by a reduced number of infected plants under field conditions. In plants resistant to virus accumulation, infection still occurs, but viral load remains relatively low. Resistance to virus movement prevents systemic spread of the virus, including its translocation into tubers. Mature plant resistance is associated with reduced potato susceptibility to a broad range of viruses, including PLRV, PVM, PVX, and PVY, at later developmental stages. In such plants, virus replication within infected tissues and subsequent systemic spread are significantly reduced. Tolerance is a form of resistance in which the virus accumulates but causes little to no symptom development. Although tolerance is a valuable trait used in a number of breeding programs, tolerant potato cultivars may also serve as reservoirs of viral infection. Resistance to aphid vectors is desirable in both potato breeding and seed tuber production because it limits virus transmission between plants. Finally, the mechanisms of most potato genes conferring resistance to viruses involve activation of signaling cascades. These signaling pathways either suppress virus accumulation in infected plants (extreme resistance) or induce a hypersensitive response. In the latter case, necrosis at the inoculation site restricts further virus replication and spread [8].

The development of potato cultivars resistant to a broad range of viral strains remains an important objective. This also applies to breeding material that combines resistance genes (R-genes) from different sources [9]. In recent years, innovative technologies such as gene editing using CRISPR and gene silencing based on RNA interference have been developed and become effective tools for controlling plant pathogens. Gene pyramiding and gene stacking are also highly innovative approaches that make it possible to combine two or more R-genes in a single genotype. These strategies have already yielded highly promising results [15,16].

A substantial number of genes conferring resistance to PVY have been identified in wild potato species [17]. Through breeding, these genes have been transferred into different potato genotypes, resulting in the development of numerous PVY-resistant cultivars. The main types of PVY-resistant genotypes carry the *Ny*, *Nc*, and *Nz* genes, associated with the hypersensitive response, and the *Ry* gene conferring extreme resistance. The hypersensitive response depends on the gene variants involved in PVY infection. Three strain-specific interactions have been described: PVYO, recognized by *Ny*; PVYc, recognized by *Nc*; and PVYz, recognized by *Nz* [18].

The development of potato cultivars with improved resistance to PVX is a classic and successful strategy in molecular phytopathology. Potato resistance to PVX is largely conferred by dominant monogenic factors introgressed from wild relatives. The *Rx1* gene was introgressed from *Solanum tuberosum* subsp. *andigena* and belongs to the *NBS-LRR* gene family [19,20]. The *Rx1* gene is located in a gene cluster on chromosome 12 and is tightly linked to *Gpa2*, which confers resistance to the pale potato cyst nematode. These traits are often inherited together in cultivated potato [21,22].

Resistance to the golden potato cyst nematode, *Globodera rostochiensis* (Wollenweber) Behrens, and the pale potato cyst nematode, *Globodera pallida* (Stone) Behrens, exhibits different inheritance mechanisms. This has important implications for breeding strategies. Breeding potato for resistance to *G. rostochiensis* has been highly successful. Most breeding programs rely on monogenic dominant resistance, which provides nearly complete protection against the most widespread pathotypes, *Ro1* and *Ro4*. The *H1* gene, located on chromosome 5, is the major resistance factor, introgressed from the wild subspecies *Solanum tuberosum* subsp. *andigena*. The H1 protein recognizes *G. rostochiensis* elicitors and triggers a hypersensitive response in roots. This response prevents syncytium development, resulting in larval starvation and death [23]. Breeding potato for resistance to *G. pallida* is considerably more challenging. Resistance is polygenic and controlled by multiple quantitative trait loci. The *Gpa2* gene is located on chromosome 12 in the same cluster as the *Rx1* gene, which confers extreme resistance to PVX. The *Gpa2* locus is associated with resistance to the *Pa2/3* pathotype and belongs to the *NBS-LRR* gene family [24,25].

Breeding potato for resistance to potato wart disease caused by the fungal pathogen *Synchytrium endobioticum* (Schilb.) Percival is of major phytosanitary importance. This pathogen is considered one of the most serious quarantine pests. Resistance to *S. endobioticum* is controlled by both monogenic and polygenic factors. Breeding is further complicated by the large number of physiological races identified within the species. More than 40 races have been reported to date. Resistance to race 1 (D1) is among the best-studied and most successful targets of potato breeding. Nearly all modern commercial potato cultivars are resistant to this race. The *Sen1* gene, located on chromosome 11, is the major dominant monogenic locus conferring resistance to race 1 (D1). The Sen1 protein triggers a local hypersensitive response, leading to necrosis of epidermal cells at sites of zoospore penetration. As a result, the pathogen is deprived of nutrients and characteristic tumors fail to develop [26,27].

Accordingly, the importance of breeding programs aimed at developing pathogen-resistant cultivars continues to increase. Another approach to solving this problem is the transition of potato seed production systems to virus-free (healthy) planting material [28,29]. An important component in the development of breeding programs for agricultural crops is the characterization of genetic variability. PCR-based methods for the detection of potato viral infections play a significant role in maintaining virus-free seed production systems [30]. The advantages of marker-assisted selection over conventional breeding approaches are evident. DNA markers make it possible to identify target genes at early developmental stages, prior to phenotypic expression. They allow researchers to rapidly and efficiently screen large amounts of breeding material, thereby significantly reducing land use and labor costs [31].

The present study pursued the following objectives:

- To assess the genetic diversity of potato accessions using PCR-based analysis;
- To identify genotypes of potential value for breeding programs focused on enhancing resistance to plant pathogens.

## 2. Materials and Methods

### 2.1. Plant Material

A total of 29 potato cultivars and 31 hybrids from the bioresource collection of the Federal Scientific Center of Agricultural Biotechnology of the Far East named after A.K. Chaiki (Ussuriysk, Russia) were evaluated during 2019–2025. The analyzed accessions were selected based on a combination of valuable breeding traits. Some hybrids were obtained through hybridization involving previously selected parental forms.

### 2.2. Sample Preparation

For molecular genetic analysis, a pooled sample of five plants per accession was used. Total DNA was isolated from green leaf tissue using a magnetic particle sorption method with the commercial reagent kit “FitoSorb” (Syntol, Moscow, Russia). To increase throughput and automation, a King Fisher Duo Prime magnetic particle processor (Thermo Fisher, Waltham, MA, USA) was employed, with the manufacturer’s protocol modified accordingly. Leaf tissue was homogenized using a Bioprep-24 bead homogenizer (Allsheng, Hangzhou, China). Subsequently, 1 mL of extraction buffer was added to the homogenate, and tubes were vigorously shaken to resuspend the material. The suspension was centrifuged at 10,000 *g* for 5 min. Then, the supernatant was transferred to new 1.5 mL microcentrifuge tubes containing 500 µL of lysis reagent. The mixture was incubated in a MSC-3000 thermoshaker (Allsheng, Hangzhou, China) at 65 °C for 10 min with intermittent shaking at 1500 rpm. After lysis, samples were centrifuged at 10,000 *g* for 10 min to pellet tissue debris. Then, 500 µL of the supernatant was transferred to a 96-well deep-well plate for purification using the magnetic particle processor (Table 1).

Nucleic acids were purified automatically according to the standard program. Total processing time was 30 min. In the first step, magnetic particles were thoroughly mixed and collected in row C wells, then transferred to row E wells. The next steps included sequential washes. In row E well, particles were mixed with wash solution 1 and transferred to row F. The procedure was then repeated in row F wells with wash solution 2, followed by transfer to row G. In row G wells, the third and final wash was performed. The magnetic particles were then removed from the solution and air-dried for 5 min. The dried particles were transferred to row A wells for elution. Elution was carried out at 65 °C under constant stirring. Afterward, the DNA-free magnetic particles were collected and transferred to row G wells. The tip comb was placed into row B wells. As a result, the purified DNA product was obtained in row A wells. DNA concentrations in the purified samples were measured using a Nano 500 spectrophotometer (Allsheng, Hangzhou, China).

### 2.3. Molecular Marker Selection

Molecular markers for the target genes conferring resistance to PVX (*Rx1*) and *G. pallida* (*Gpa2*), as well as the corresponding primers, were selected from [32]. The PVX marker for the *Rx1* gene, Gpa2-2 for the *Gpa2* gene, and NL 25 for the *Sen1* gene were used (Table 2). PCR conditions were optimized by adjusting reaction mixture composition using positive controls, which consisted of DNA from potato cultivars known to carry the target genes. The following cultivars were used for method calibration: Meteor (*Rysto*, *Rx1*, *Sen1*, *Gpa2*, *H1*), Vektor (*Rx1*, *Gpa2*), Yubilyar (*Gpa2*), and Zhokovsky ranniy (*Gpa2*, *Rx1*) [33,34,35].

### 2.4. PCR Analysis

PCR was performed in a 10 μL reaction volume using 2X BioMaster HS-Taq PCR-Color mix (Biolabmix, Novosibirsk, Russia) on a T100 thermal cycler (Bio-Rad, Hercules, CA, USA). Each reaction contained 10–50 ng of template DNA. The thermal cycling conditions for multiplex PCR were applied according to Saynakova et al. (2018) [35]. All reactions were set up individually.

PCR products were separated by electrophoresis in 2% agarose gels stained with ethidium bromide. DNA fragments were visualized under UV irradiation using a Gel-Doc XR + gel documentation system (Bio-Rad, Hercules, CA, USA). Fragment lengths were determined using the Step 50 Plus and Step 100 markers (Biolabmix, Novosibirsk, Russia). The presence of target markers was determined by comparison with the positive controls carrying fragments of known length.

### 2.5. Statistical Analysis

The contingency of resistance loci and linkage disequilibrium were analyzed using nonparametric methods for contingency table analysis. *p*-values for pairwise associations were calculated using two-tailed Fisher’s exact test or Pearson’s chi-squared test. Association significance was indicated as follows: *ns*—not significant (*p* ≥ 0.05); *—*p* < 0.05; ***—*p* < 0.001), according to conventional biometric protocols [36]. Heatmaps were generated in TBtools v.2.0 (SCAU, Guangzhou, China) [37]. Narrow-sense heritability (*h*^2^) for qualitative molecular markers were estimated by linear regression [38]. This parameter represents the proportion of total phenotypic variation in a population attributable solely to additive genetic effects [39]. The validity of the results was assessed by one-way ANOVA followed by Fisher’s least significant difference post hoc test (*LSD*), using MS Excel 2007 and Statistica 10 (StatSoft, Inc., Tulsa, OK, USA). Means (*M*) and *t*_0_._05_ ½SEM were calculated.

## 3. Results and Discussion

### 3.1. Calibration of the PCR-Based Identification Method for the 57R, N195, and YES3A Markers Linked to the Genes Conferring Resistance to G. Rostochiensis (H1) and PVY (Rysto)

Currently, breeding programs require comprehensive databases that combine phenotypic data on breeding material with information on DNA markers. The latter should be associated with QTLs controlling economically important traits. Over the past two decades, numerous DNA markers have been developed for potato, enabling marker-assisted selection (MAS) of promising genotypes. These markers are now widely used in molecular screening globally [23,40].

Many agronomically important potato traits are monogenic, including resistance to pests and pathogens. These include resistance to late blight caused by *Phytophthora infestans* (Mont.) de Bary, PVX, PVY, PVS, PLRV, cyst nematodes, and *S. endobioticum*. Molecular markers linked to specific R-genes are effective tools for accelerating breeding. Among these are the *Rpi* genes conferring resistance to late blight; the *Rysto*, *Ryadg*, and *Rychc* genes associated with immunity to PVY; the *Rx1* gene conferring resistance to PVX; the *H1* and *Gro1-4* genes linked to resistance to *G. rostochiensis*; and the *Gpa2* gene associated with resistance to *G. pallida* [41].

**Figure 1:**
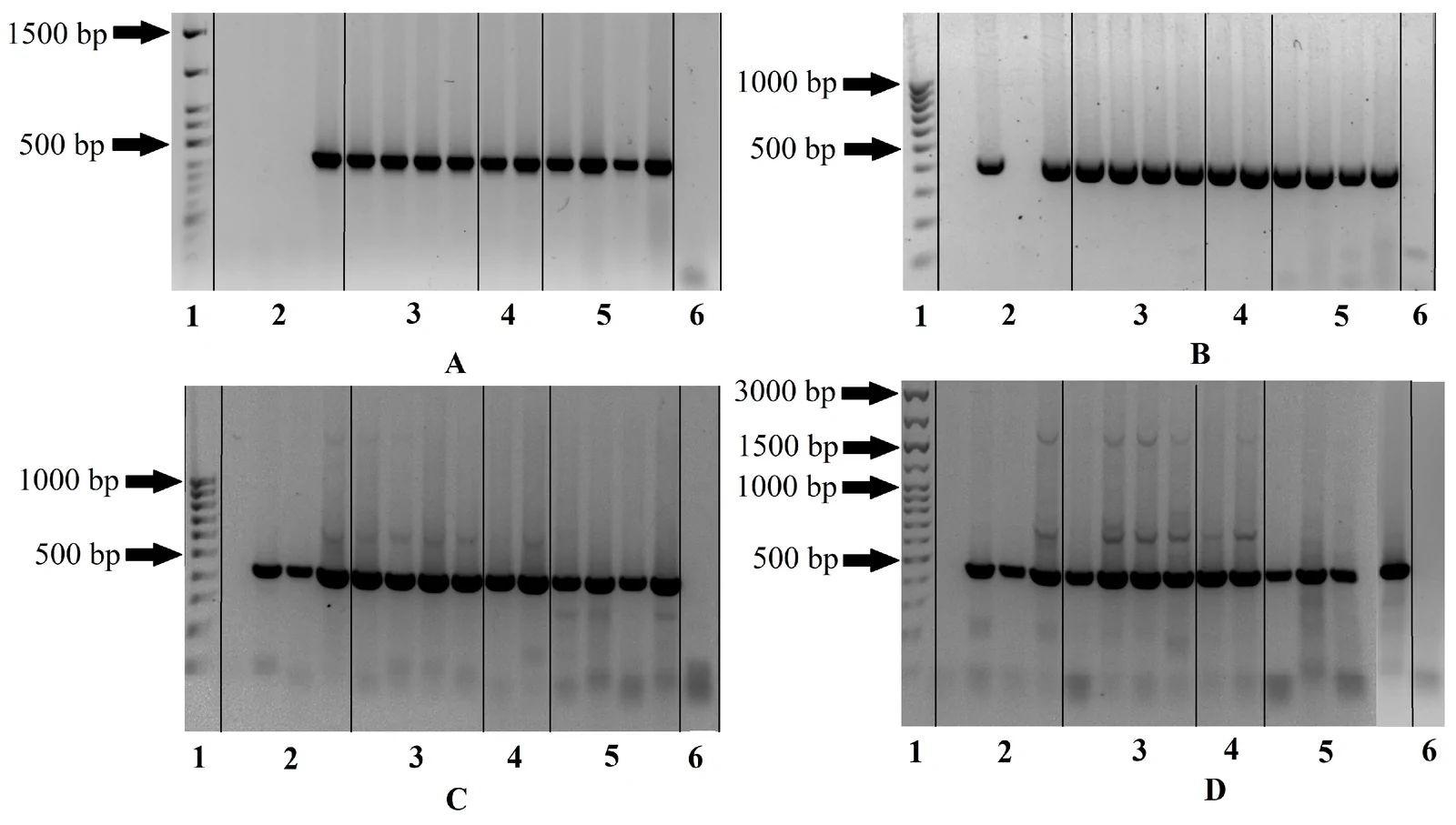
Electrophoretic profiles of PCR products obtained from positive controls carrying the Gpa2-2 marker linked to the *Gpa2* gene at different magnesium chloride concentrations: (**A**)—0.5 mM, (**B**)—1.0 mM, (**C**)—2.0 mM, (**D**)—2.5 mM. Lanes: 1—fragment length markers, 2—Zhukovsky ranniy, 3—Yubilyar, 4—Vektor, 5—Meteor, 6—negative control (H*_2_*O).

**Figure 2:**
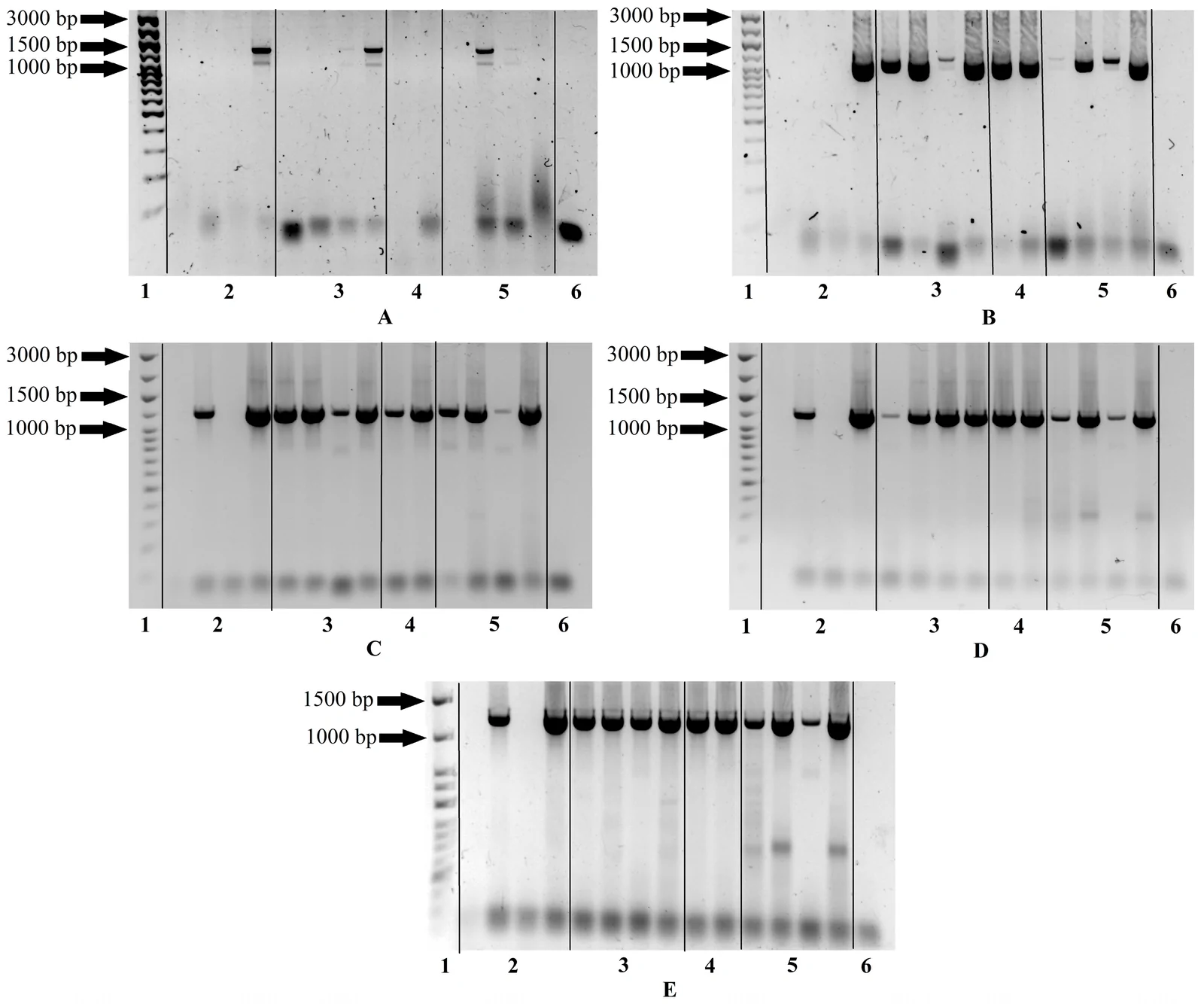
Electrophoretic profiles of PCR products obtained from positive controls carrying the PVX marker linked to the Rx1 gene at different magnesium chloride concentrations: (**A**)—0.5 mM, (**B**)—1.0 mM, (**C**)—1.5 mM, (**D**)—2.0 mM, (**E**)—2.5 mM. Lanes: 1—fragment length markers, 2—Zhukovsky ranniy, 3—Yubilyar, 4—Vektor, 5—Meteor, 6—negative control (H_2_O).

In 2019, PCR-based detection of the 57R and N195 markers and the YES3A marker was optimized. The first two are linked to the *H1* gene (*G. rostochiensis* resistance), while YES3A is linked to the *Rysto* gene (PVY resistance). In 2020, detection conditions were optimized for markers linked to potato genes conferring resistance to *G. pallida* and PVX. As a result, the optimal concentrations of magnesium chloride were determined: 2.0 mM for *Gpa2* (Figure 1) and 2.5 mM for *Rx1* (Figure 2). The thermal cycling conditions for multiplex PCR were applied according to Rogozina E.V. et al., 2019 [34]; all reactions were set up individually.

### 3.2. Identification of Pathogen Resistance Donors Among the Studied Potato Cultivars

Accessions should be annually screened for agronomically important traits, including resistance to pests and pathogens [23]. This approach enables the identification of donor cultivars resistant to viruses (PVX and PVY) and cyst nematodes (*G. pallida* and *G. rostochiensis)* [42]. These studies were conducted over 2019–2023.

**Figure 3:**
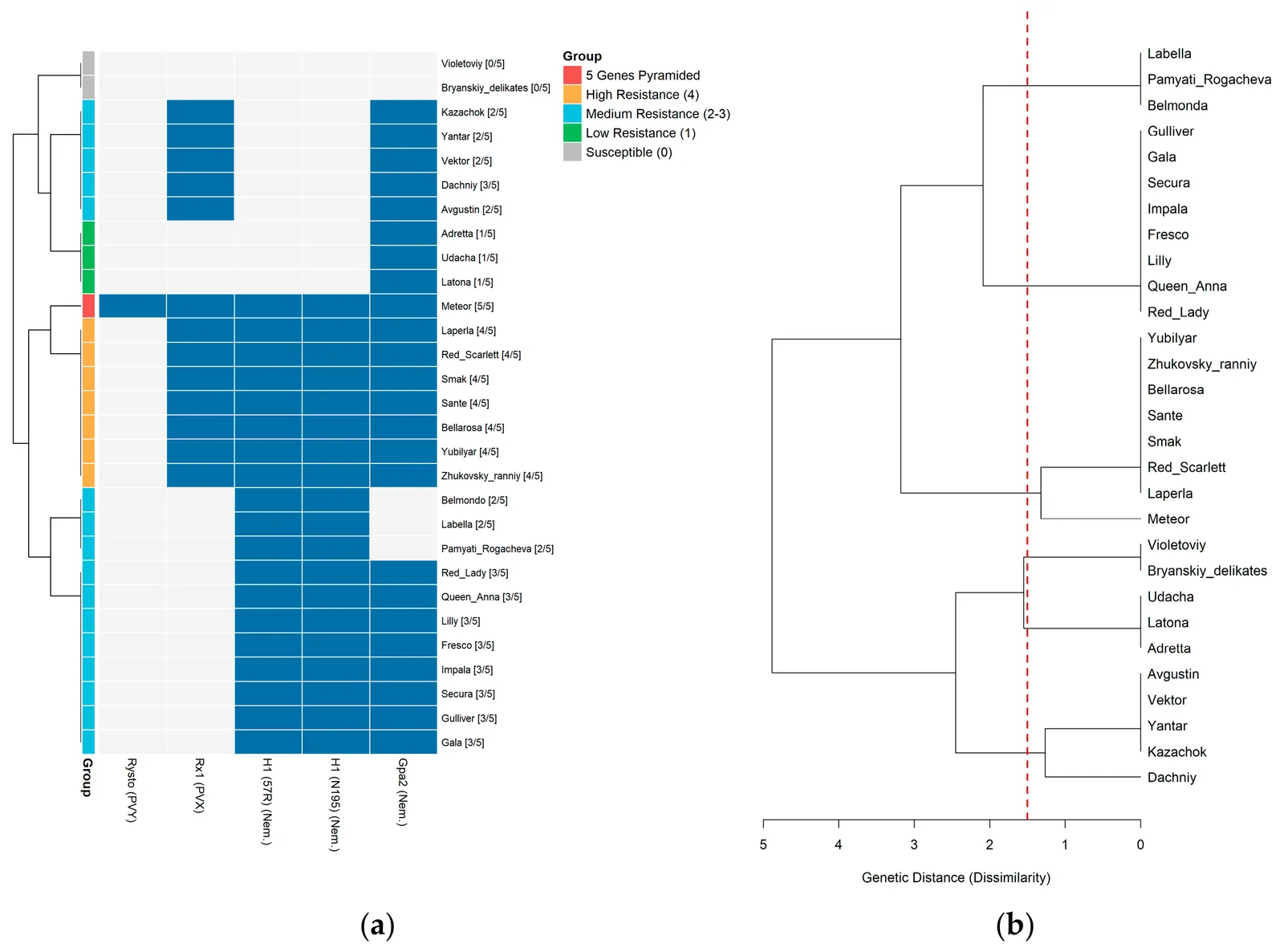
(**a**) Heatmap showing the distribution of resistance-associated genetic markers across 29 analyzed potato accessions. (**b**) Dendrogram of genetic similarity among 29 analyzed potato accessions based on molecular markers of R-genes.

A total of 29 potato cultivars were genotyped for five key resistance loci: *Rysto* (resistance to PVY), *Rx1* (resistance to PVX), *H1* (the *H1* (57R) and *H1* (N195) loci associated with resistance to *G. rostochiensis*), and *Gpa2* (resistance to *G. pallida*). Based on similarities in allelic composition, the analyzed accessions were grouped in several clusters (Figure 3a). Cultivar Meteor carried the complete set of target markers (5/5). The *Rysto* locus makes this cultivar especially valuable for breeding programs, as it confers extreme resistance to PVY and was absent in the remaining accessions. Cultivar Meteor therefore formed the polyresistant core of the analyzed collection. Cultivars Yubilyar, Zhukovsky ranniy, Bellarosa, Sante, Smak, Red Scarlett, and Laperla formed a stable highly resistant cluster [4/5], combining resistance to PVX (*Rx1*) with complex resistance to both cyst nematode species. Although these cultivars lacked the *Rysto* locus, they should be considered priority genotypes for further introgression of virus resistance traits. Visual analysis demonstrated a high frequency of the *H1* (57R) and *H1* (N195) loci. This combination was detected in 19 cultivars, confirming their widespread use in modern breeding programs focused on nematode resistance. Cultivars Bryanskiy delikates and Violetoviy formed a separate cluster lacking all tested dominant markers. Such a result highlights the need to involve both genotypes in hybridization programs aimed at improving their resistance potential.

Visualization of the obtained data confirmed substantial genetic heterogeneity within the analyzed collection and allowed identification of the most promising parental forms for marker-assisted selection. The observed visual pattern also indicated genetic linkage or joint selection of several markers. In particular, the *H1* (57R) and *H1* (N195) loci showed a high degree of co-segregation and co-occurred in 90% of the analyzed genotypes. In contrast, the *Rysto* locus appeared sporadically (3.4%) and demonstrated an independent distribution pattern. This finding suggests the limited use of *Rysto* in the current breeding process within the analyzed cultivar group. The structure of genetic diversity was characterized by pronounced asymmetry in the frequency distribution of individual markers.

UPGMA (Unweighted Pair Group Method with Arithmetic Mean) has repeatedly demonstrated its effectiveness in differentiating collection and breeding material based on PCR-markers of R-genes. In particular, large-scale studies [43] have used the RYSC3 and YES3-3B DNA-markers for screening PVY resistance loci. This approach enabled highly accurate structuring of cultivar diversity and identification of hidden duplicates within the gene pool. Genotypes carrying markers of extreme resistance to viruses (e.g., *Ry* or *Rx1* locus) and nematode resistance (*H1* and *Gpa2* genes) have been observed to cluster within the same group. This indicates successful pyramiding of R-genes during the breeding process. Such gene combinations existing within a single cluster represent valuable donor material for further hybridization [44,45].

Hierarchical cluster analysis was performed to evaluate phylogenetic relationships among 29 potato accessions according to resistance-associated loci (Figure 3b). Clustering was based on binary SSR-profiling matrices generated using primers for diagnostic markers. Genetic divergence was assessed using dissimilarity distance values ranging from 0 to 5 along the *x*-axis. To verify discrete cluster groups, a critical cut-off threshold of 1.5 was established and indicated by a red dashed line. At the highest hierarchical level, the dendrogram topology clearly separated the analyzed collection into two independent major pools (megaclusters) at a dissimilarity value of ≈4.8. This pattern indicates substantial divergence among the studied cultivars in the presence or absence of analyzed resistance alleles. At a cut-off threshold of 1.5, the analyzed set of sixteen Russian and foreign cultivars was separated into three subclusters. The first subcluster included cultivars Labella, Pamyati Rogacheva, and Belmonda, which showed a high degree of homology with a minimum genetic distance of <0.6. This indicates identical marker profiles for the *Rx1*, *Gpa2*, and *Sen1* genes. The second subcluster formed a dense group of eight cultivars: Fresco, Gulliver, Gala, Impala, Secura, Lilly, Queen Anna, and Red Lady. The low level of intracluster dissimilarity suggests a narrow genetic basis for the analyzed loci, likely reflecting the use of common parental lines in their pedigrees. The third subcluster consisted of cultivars Yubilyar, Zhukovsky ranniy, Bellarosa, Sante, and Smak. They grouped within a common node at a threshold of 1.3 and were characterized by a similar SSR-marker distribution pattern.

The lower cluster comprised 13 genotypes and showed a more heterogeneous structure. At a dissimilarity level of 1.5, it split into five genetic lineages. Cultivars Red Scarlett, Laperla, and Meteor formed a separate node at a threshold of 1.3. In contrast, cultivars Violetoviy and Bryanskiy delikates showed stable marker similarity, forming a pair with the minimum linkage distance (≈0.2). This indicates complete duplication of allelic composition across the target loci. Cultivars Udacha, Latona, and Adretta (node at approximately 1.1) exhibited a high degree of homology in their marker profiles. Cultivars Avgustin and Vektor represented distinct genotypes and showed the highest divergence within the lower megacluster, separating at early hierarchical levels (distance > 2.4). This pattern suggests a unique SSR-profile, potentially reflecting a specific *Rx1*/*Gpa2*/*Sen1* gene combination or the absence of most target loci, clearly distinguishing them from the remaining cultivars. The final cluster consisted of cultivars Yantar, Kazachok, and Dachniy (linkage node ≈ 1.2). Their grouping within a single subcluster occurred at a defined level of genetic dissimilarity, indicating a high degree of isomorphism in their PCR-profiles.

The observed separation of cultivars into distinct clusters reflects differences in the enrichment of the analyzed gene pool with target R-genes. High clustering density within certain groups indicates a high degree of relatedness based on the analyzed genes: *Rx1*, *Gpa2*, and *Sen1*. For example, high density was observed in the cluster consisting of cultivars Fresco, Gulliver, Gala, Impala, Secura, Lilly, Queen Anna, Red Lady and in Violetoviy/Bryanskiy delikates cultivar pair.

**Figure 4:**
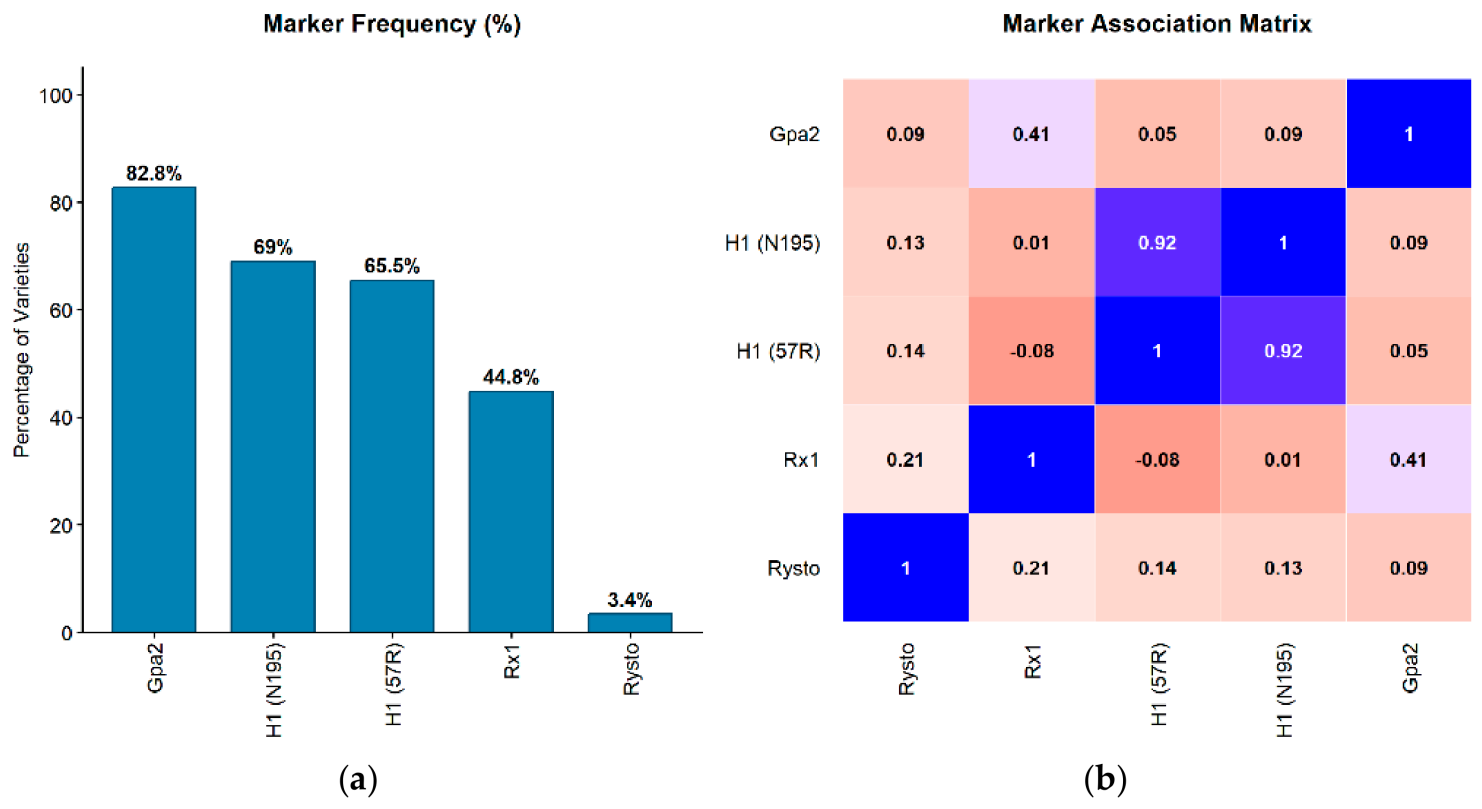
Frequency distribution of molecular markers (**a**) and evaluation of their linkage or joint association based on contingency coefficients (**b**).

In the studied population, the *Gpa2* (82.8%) locus and the *H1* gene group (65.5–69.0%) were predominant. This highlights the importance of these genomic regions in modern potato breeding programs. The *Rysto* locus showed the lowest frequency (3.4%), confirming the limited availability of sources with broad virus resistance in the analyzed collection. Correlation analysis revealed a strong positive association between the *H1* (57R) and *H1* (N195) loci (r = 0.86). This finding likely reflects their close genetic linkage or origin from a common ancestor. A significant association was also observed between the *Rx1* and *Gpa2* loci (r = 0.54), suggesting their frequent co-occurrence in the studied cultivars. Negative or near-zero correlations between the *Rysto* gene and the remaining genes confirmed its independent inheritance and unique position within the collection structure (Figure 4a,b).

**Figure 5:**
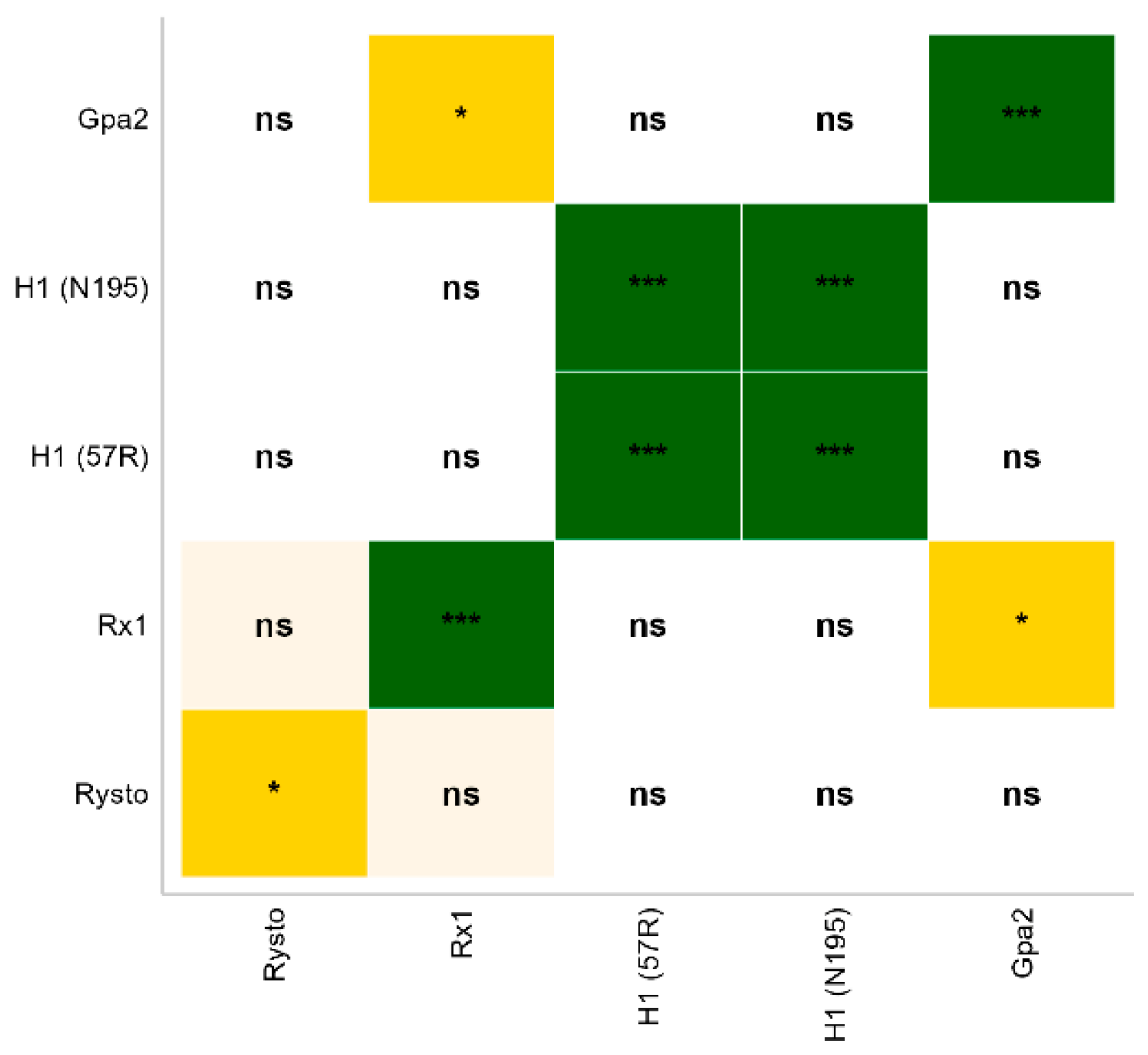
Evaluation of non-random co-occurrence of resistance loci based on significance testing (*p*-value).

Joint contingency analysis of resistance traits was performed to identify genetic linkage or non-random associations among the studied loci. Analysis of the correlation structure revealed a strong and highly significant association between the *H1* (57R) and *H1* (N195) loci (*p* < 0.001 ***). Symmetrical reciprocal blocks of high significance indicate that these resistance loci are either inherited codominantly or remain in strong genetic linkage (Figure 5).

This finding confirms their common breeding target, namely resistance to the *Po1* and *Po4* pathotypes of *G. rostochiensis*. A strong statistically significant association was detected between the *Rx1* gene conferring resistance to PVX and the *Gpa2* gene conferring resistance to *G. pallida* (*p* < 0.001, from * to ***). Significant cross-associations likely reflect either clustering of these genes on the same chromosome or their coordinated selection during breeding for group resistance. The *Rysto* gene associated with resistance to PVY showed the highest degree of autonomy within the studied collection. Weak statistical significance (*p* < 0.05, *) was observed only in association with its own orthogonal distribution and in combination with the *Gpa2* locus. Most other paired gene combinations were statistically non-significant (ns, *p* ≥ 0.05). This indicates independent segregation of these loci within the populations and the absence of strong selection pressure.

The obtained results enable optimization of PCR screening. Strong linkage between *H1* (57R) and *H1* (N195) makes it possible to exclude one of the primers, thereby reducing large-scale analysis cost without loss of accuracy. In contrast, the independent distribution pattern of *Rysto* requires separate PCR testing of this locus when developing donors with complex resistance.

### 3.3. Use of Selected Potato Donor Cultivars in Breeding

The use of contrasting parental components remains a classical and highly effective approach to achieving heterosis and segregation of polygenic traits in hybrid populations [43]. Molecular profiling of modern cultivars has shown that their hybridization enables effective R-gene pyramiding. Potato cultivars may be developed by combining extreme virus resistance loci (e.g., *Ry* and *Rx1*) with nematode resistance factors (*H1*, *Gpa*) in a single hybrid. Long-term MAS practice confirms that this approach minimizes the risk of immunity breakdown under changing nematode races and emerging recombinant PVY strains, such as PVY^NTN^ [41].

A genetically diverse set of seven Russian and foreign cultivars was used as parental material for developing hybrids with predetermined traits in 2021–2025: Adretta, Gala, Sante, Smak, Utro, Zhukovsky ranniy, and Yantar. These genotypes were selected based on differences in R-gene architecture and the presence of economically important traits.

Sante is a classical Dutch cultivar, serving as a donor of complex resistance, with a growing period of 100–110 days. It carries a broad spectrum of R-genes, including loci associated with resistance to viruses and nematode species. All these factors make Sante a valuable component in crossing combinations aimed at gene pyramiding [46].

Zhukovsky ranniy is a Russian cultivar characterized by high plasticity. It combines very early maturity (80–90 days) with genetically determined resistance to biotic stressors. Its placement within the same cluster as cultivar Sante reflects similarity of their marker profiles [47].

Smak is a late-maturing potato cultivar (110–120 days) developed at the Federal Scientific Center of Agricultural Biotechnology of the Far East named after A.K. Chaiki. It showed a high degree of topological homology with the Sante/Bellarosa group. Smak is often used as a parent in hybridization due to stable expression of target resistance alleles, confirmed by SSR analysis [48].

Adretta, a well-known German cultivar with a growing period of 100–110 days, forms a stable core together with Udacha and Latona. In breeding programs, it serves as a source of a high nutritional and culinary quality and field resistance to late blight. Its substantial genetic distance from cultivars of the first cluster makes Adretta a priority genotype for top crosses [49].

Yantar is another potato late-maturing cultivar (110–120 days) developed at the Federal Scientific Center of Agricultural Biotechnology of the Far East named after A.K. Chaiki. It forms a separate terminal node with cultivar Kazachok. The specific SSR profile of Yantar reflects a unique allelic combination that can reduce undesirable correlations between traits and expanding allelic diversity in hybrid populations [50].

Utro is a Russian cultivar characterized by high adaptability to unfavorable soil and climatic conditions and early maturity (85–90 days). It exhibits a marker pattern independent of the upper megacluster, which determines its high combining ability in crosses with donors such as Gala or Labella [51].

Gala is a highly productive German cultivar with a growing period of 90–100 days. It is often used as a key component in breeding programs aimed at improving potato culinary quality. In the dendrogram topology, Gala forms a dense cluster together with Gulliver, Secura, Impala, and others. The low level of intracluster dissimilarity within this group indicates a narrow genetic basis for the analyzed loci (*Rx1*, *Gpa2*, and *Sen1*). In hybridization programs, Gala serves as a valuable donor of tuber marketability, nest uniformity, and resistance to mechanical injury. However, it requires carefully selected contrasting partners from the lower megacluster to compensate for the limited allelic diversity of the analyzed loci [49].

The remaining 22 cultivars did not form flowers, and their pollen fertility was insufficient, making their involvement in hybridization unfeasible.

### 3.4. Analysis of the Obtained Hybrid Combinations for Inheritance of Parental R-Genes

Using combinatory breeding, we developed 10 successful potato hybrid combinations and identified accessions with different numbers of R-gene markers. Most hybrid combinations were characterized by a uniform distribution of target molecular markers within the progeny.

**Figure 6:**
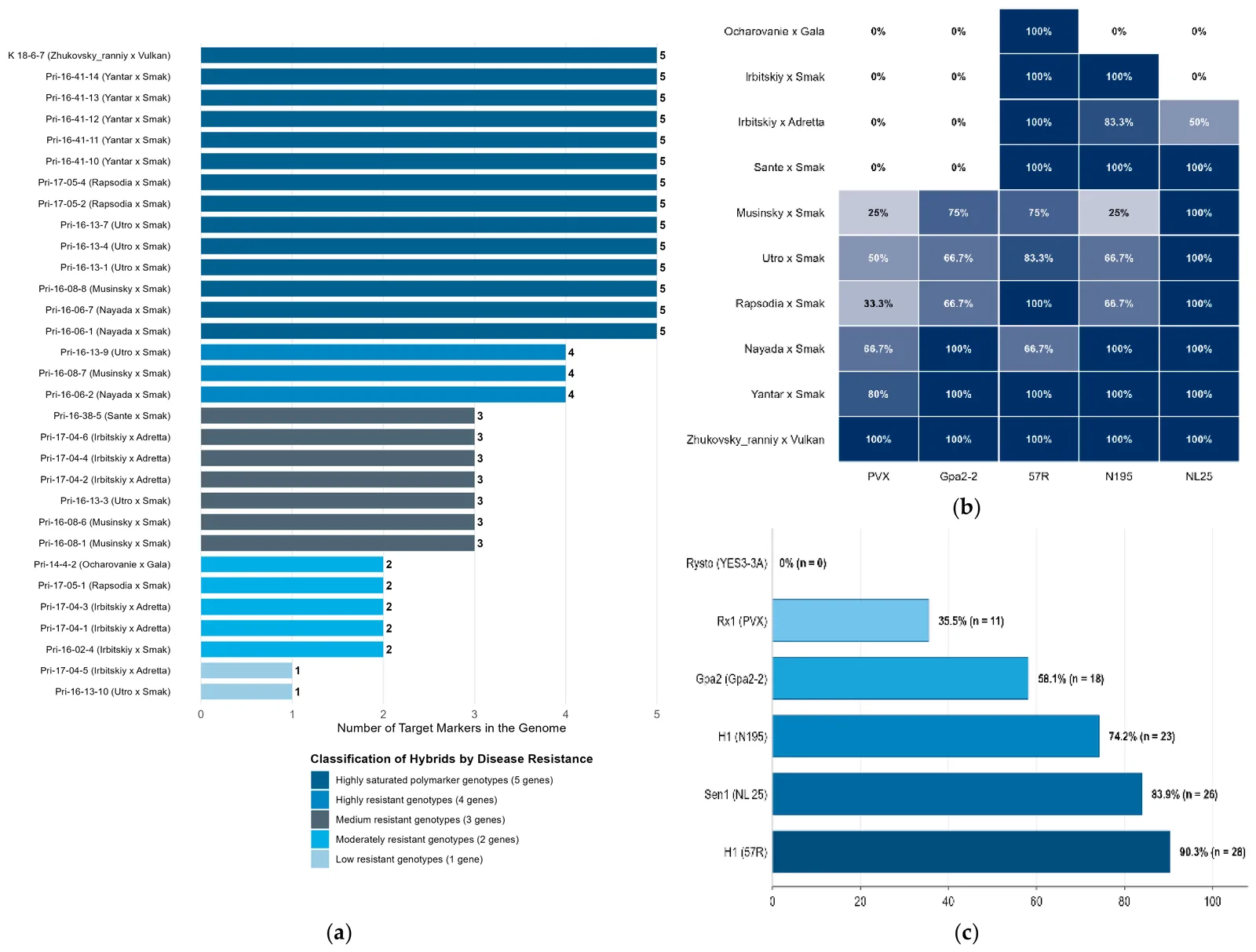
(**a**) Genetic profiles of potato hybrid progeny enrichment (*n* = 31) with target marker alleles associated with resistance: 1—PVX; 2—Gpa2-2; 3—57R; 4—N195; and 5—NL25. (**b**) Combining ability profile of parental pairs based on the effectiveness of target DNA-marker transmission to hybrid progeny. (**c**) Effectiveness of allele accumulation associated with resistance to viruses and nematode species within the pool of potato hybrid progeny.

To evaluate the effectiveness of the target gene transmission in crosses, 31 potato hybrid lines were ranked. The ranking was based on the cumulative accumulation of molecular markers associated with resistance to plant pathogens (Figure 6a). This parameter reflects the total number of target resistance markers simultaneously detected in a single genotype from the six analyzed markers: PVY, PVX, Gpa2-2, 57R, and N195.

The YES3A marker was not detected in any analyzed genotype. The absence of the dominant *Rysto* gene, which confers extreme resistance to PVY, may have several causes. This finding has been reported in multiple molecular-genetic screenings worldwide [52,53]. The *Rysto* gene originates from the wild tetraploid species *Solanum stoloniferum*, native to Mexico [54]. Unlike cultivated potato, this species differs in ploidy and has strong reproductive barriers. Introgression into breeding lines therefore requires a multistep compatibility-breaking procedures, such as polyploidization or backcrossing. This has historically limited the number of donor cultivars. In Europe, the dominant *Ryadg* gene, introgressed from *Solanum tuberosum* subsp. *andigena*, has been used for PVY resistance. This subspecies crosses readily with modern potato cultivars without intermediate biotechnological steps. Most PVY-resistant cultivars carry *Ryadg*, detected with the RYSC3 marker, whereas *Rysto* remains rare [52].

The studied hybrid population exhibited pronounced divergence and continuous variability in marker allele enrichment. Values ranged from one to five markers, which were the minimum and maximum detected in the analyzed collection. Based on the obtained genotypic profiles, the breeding material was classified into three discrete groups. The first group included 14 hybrid combinations represented by polymarker genotypes carrying five target resistance markers and therefore possessing the highest breeding potential. Within this group, the hybrid family derived from the Yantar × Smak cross and represented by five related lines (Pri-16-41-14, Pri-16-41-13, Pri-16-41-12, Pri-16-41-11, and Pri-16-41-10) was particularly predominant. This pattern indicates high combining ability of the parental pair and suggests synergistic interactions during the transmission of resistance alleles. High effectiveness of gene pyramiding was also observed in the following combinations: Rapsodia × Smak (lines Pri-17-05-4 and Pri-17-05-2), Utro × Smak (Pri-16-13-7, Pri-16-13-4, and Pri-16-13-1), Nayada × Smak (Pri-16-06-7 and Pri-16-06-1). The Zhukovsky ranniy × Vulkan and Musinsky × Smak combinations were each represented by a single polymarker genotype, namely the K 16-6-7 and Pri-16-08-8 hybrids, respectively. The high frequency of hybrids carrying five markers simultaneously in crosses with Smak confirms its high effectiveness as a donor of polygenic resistance.

Genotypes carrying three to four markers were classified as an intermediate segregation group. This group reflects intermediate stages of meiotic segregation and independent recombination of parental chromosomes. Genotypes carrying four markers and exhibiting balanced genetic architecture are promising for further breeding aimed at trait fixation, including Pri-16-13-9 (Utro × Smak), Pri-16-08-7 (Musinsky × Smak), and Pri-16-06-2 (Nayada × Smak). Genotypes carrying three markers included lines derived from the Irbitskiy × Adretta cross (Pri-17-04-6, Pri-17-04-4, and Pri-17-04-2), as well as individual seedlings from the Utro × Smak (Pri-16-13-3) and Musinsky × Smak (Pri-16-08-6 and Pri-16-08-1) families, and the Pri-16-38-5 hybrid derived from the Sante × Smak cross.

The low-enrichment group consisted of genotypes carrying the minimum number of markers, having lost most dominant resistance alleles during segregation. The following accessions carried two markers: Pri-17-04-3 and Pri-17-04-1 (Irbitskiy × Adretta), Pri-14-4-2 (Ocharovanie × Gala), Pri-17-05-1 (Rapsodia × Smak), and Pri-16-02-4 (Irbitskiy × Smak). The lowest level of genetic enrichment (one marker) was observed in the Pri-17-04-5 (Irbitskiy × Adretta) and Pri-16-13-10 (Utro × Smak) seedlings. These results indicate an almost complete elimination of the target parental resistance loci in the analyzed accessions.

The identified five-marker hybrids are characterized by potential polygenic resistance to a broad spectrum of pathogens, including PVX, PVY, cyst nematode species, and *S. endobioticum*. These polymarker accessions can be recommended for use in breeding programs as valuable genetic donors.

Ten hybrid families were analyzed using a matrix of hybridization effectiveness based on the frequency of the target molecular markers (Figure 6b). As a result, the combining ability of parental combinations was evaluated and the most effective genetic donors of resistance to viruses and nematode species were identified. Analysis of the frequency profiles suggested a high degree of heterozygosity in the original breeding material. This value was reflected by a wide range of variability in allele transmission frequency, from complete elimination of loci to 100% fixation in the progeny.

The Zhukovsky ranniy × Vulkan combination showed the highest effectiveness of trait transmission, exhibiting a 100% transmission frequency for all five analyzed markers (PVX, Gpa2-2, 57R, N195, and NL25). Such high effectiveness suggests that dominant resistance alleles in the parental pair were homozygous, ensuring inheritance of complex resistance factors in all seedlings. Hybrid combinations involving cultivar Smak exhibited high combining ability, particularly the Yantar × Smak and Nayada × Smak crosses. Complete fixation (100.0%) was observed for markers associated with resistance to nematode species (Gpa2-2 and N195) and *S. endobioticum* (NL25). In contrast, the transmission frequency of the STS marker linked to the PVX resistance gene reached 60.0% and 66.7%, respectively. The observed segregation at the PVX marker suggests that one of the parental genotypes was heterozygous for this R-gene.

The Rapsodia × Smak and Utro × Smak families represented classical examples of independent segregation of resistance loci during meiosis. In these crosses, the NL25 marker showed a 100% transmission frequency, whereas continuous segregation was observed for the remaining markers. The transmission frequency of the 57R marker reached 100% in the Rapsodia × Smak cross and 83.3% in Utro × Smak. Transmission frequencies of the Gpa2-2 and N195 markers ranged from 66.7 to 83.3%, approximating the expected monohybrid segregation ratio (3:1) for crosses between heterozygous parents. The PVX marker was transmitted at a frequencies of 33.3% and 50.0%, consistent with the expected 1:1 segregation ratio in a test cross.

The Musinsky × Smak family was of particular interest. Intermediate segregation ratios were observed for four of the five analyzed markers: 25.0% for PVX and N195, 75.0% for Gpa2-2 and 57R. This segregation distortion may indicate linkage disequilibrium between the analyzed loci or selective advantages of gametes carrying alternative alleles.

The Sante × Smak, Irbitskiy × Adretta, Irbitskiy × Smak, and Ocharovanie × Gala crosses exhibited complete elimination (0%) of the PVX and Gpa2-2 markers. Direct selection for these resistance factors within the corresponding progenies is unfeasible due to the absence of dominant alleles in the parental forms. Nevertheless, these combinations remain valuable as donors of individual resistance factors. The 57R marker was fixed (100%) in all four families, suggesting homozygosity of the parental genotypes for this marker. Complete inheritance of the N195 marker (100%) was observed in combinations involving cultivars Smak and Sante. In contrast, in the Irbitskiy × Adretta combination, the transmission frequency reached 83.3%. The NL25 marker associated with resistance to *S. endobioticum* was transmitted at a high frequency in the Sante × Smak (100%) and Irbitskiy × Adretta (50.0%) crosses. However, this allele was completely eliminated in the Irbitskiy × Smak and Ocharovanie × Gala families.

Matrix screening accurately confirmed the donor potential of the original breeding material. The Zhukovsky ranniy × Vulkan, Yantar × Smak, and Nayada × Smak combinations exhibited the highest combining ability and ensured effective gene pyramiding in the progeny.

Markers of the *H1* gene associated with resistance to *G. rostochiensis* were the most prevalent in the hybrid population (Figure 6c). The *H1* (57R) locus exhibited the highest transmission frequency in the population. It was detected in 90.3% of the analyzed hybrids, corresponding to 28 out of 31 hybrid lines. The *H1* (N195) locus showed a similarly high transmission frequency of 74.2% (*n* = 23). The high enrichment of the population with *G. rostochiensis* resistance factors can be explained by the involvement of homozygous *H1* donors in the crosses (e.g., cultivars Smak, Sante, and Adretta). The SCAR marker NL 25 linked to the *Sen1* gene conferring resistance to *S. endobioticum* belonged to the same category. Its detection frequency reached 83.9% (*n* = 26).

Loci associated with resistance to *G. pallida* and PVX showed intermediate frequencies in the population. The *Gpa2* (Gpa2-2) locus was detected in 58.1% (*n* = 18) of the analyzed genotypes. Its moderate frequency likely reflects the heterozygous state of this gene in many parental forms, resulting in classical Mendelian segregation of the trait in the progeny. The *Rx1* (PVX) locus was detected at a lower, although still breeding-relevant, frequency of 35.5% (*n* = 11). The relatively low occurrence of this marker suggests the predominance of test-cross-like combinations, in which the dominant PVX resistance allele was contributed by only one heterozygous parent. In addition, partial elimination of PVX-positive gametes during microsporogenesis cannot be excluded.

The *Rysto* (YES3-3A) locus was not detected in the analyzed progeny. The absence of this locus indicates that none of the parental genotypes involved in hybridization carried the dominant *Rysto* allele. This finding highlights the need to incorporate additional genetic sources (e.g., cultivars Meteor, Bellarosa, and Yubilyar) to introduce PVY resistance into breeding lines.

**Figure 7:**
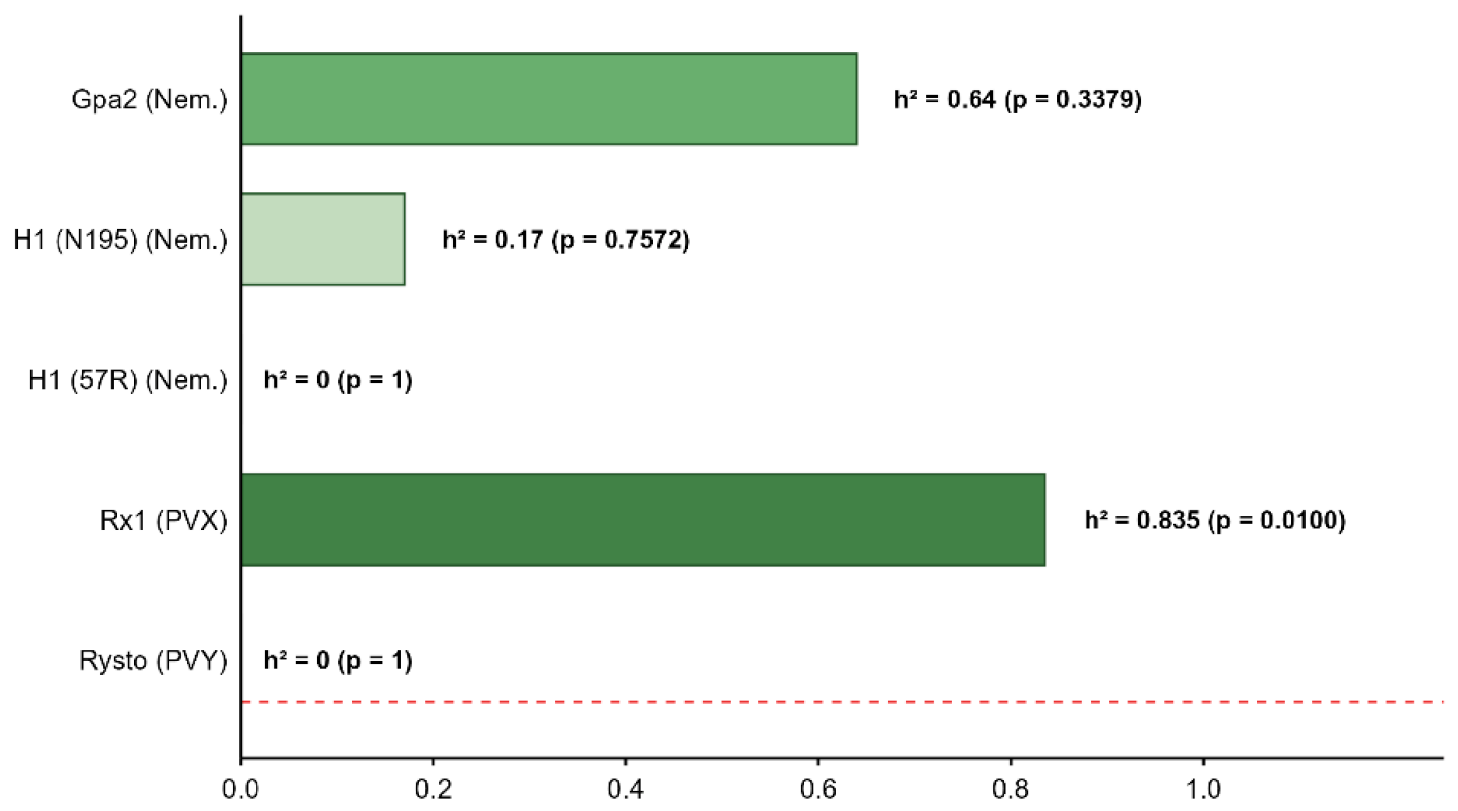
Differentiation of resistance loci according to additive genetic variance and significance levels of (*p*-values) linear regression model.

Biometric evaluation of narrow-sense heritability coefficients (*h*^2^) was performed for the five analyzed resistance loci. The aim was to estimate the proportion of genetic variation determined by additive gene effects and to predict the potential rate of breeding progress (Figure 7).

The STS marker of the *Rx1* gene exhibited the highest heritability value. The coefficient reached *h*^2^ = 0.835, with strong statistical significance in the regression model (*p* = 0.01). This value exceeded the critical threshold of breeding reliability (*h*^2^ > 0.7), indicating that the trait was predominantly controlled by additive genetic effects. The close correspondence found between the expected parental genotypes and the observed marker expression in the progeny confirms the high effectiveness of direct mass selection for this locus at the earliest hybridization stages. The *Gpa2-*associated marker showed moderate breeding value (0.3 ≤ *h*^2^ ≥ 0.7), although the regression model itself was characterized by low statistical significance (*p* = 0.3379). The *h*^2^ value of 0.64 indicates that up to 64% of variation in marker transmission frequency was determined by additive effects contributed by the parents. However, the elevated *p*-value suggests that the sample size of the hybrid families should be increased to achieve statistical reliability. Therefore, individual selection for this locus should be combined with obligatory progeny evaluation. The *H1* (N195) locus exhibited a low heritability coefficient (*h*^2^ = 0.17) and lacked statistical significance (*p* = 0.7572). Such a low value indicates that variation in transmission frequency of this marker among hybrid progenies depends only weakly on the average parental characteristics. Instead, transmission frequency is largely determined by overdominance effects or specific combining ability of particular parental pairs. Under these conditions, direct selection based on phenotype or marker presence is inefficient. The *H1* (57R) and *Rysto* loci exhibited a heritability coefficient of zero. However, from a genetic perspective, the causes of these zero values were fundamentally different. From the *H1* (57R) locus, the zero coefficient resulted from near-complete fixation in the hybrid population (occurrence frequency approaching 100%), which effectively eliminated trait variance. In contrast, for the *Rysto* locus, the zero value was caused by the complete absence of the allele in the population (occurrence frequency approaching 0%). In both cases, calculation of the linear regression model was not feasible because of trait monomorphism, making selection within the analyzed population ineffective.

## 4. Conclusions

This study demonstrated that the structure of genetic diversity was characterized by pronounced asymmetry in the frequency distribution of individual markers. The *Gpa2* locus was the most widespread in the analyzed population, with a frequency of 82.8%. This indicates its successful long-term incorporation into the gene pool of modern potato cultivars. The *H1* (N195) and *H1* (57R) loci associated with resistance to nematode species were also widely represented. They occurred in 69.0% and 65.5% of the analyzed genotypes, respectively. The high frequency of these genes reflects the long-standing breeding focus on resistance to *G. rostochiensis*. The *Rx1* gene conferring resistance to PVX was detected in 44.8% of the studied accessions, indicating a moderate distribution of this resistance factor within the collection. This enabled the differentiation of the material into two nearly equal groups according to PVX resistance. In contrast, the *Rysto* gene was detected at a critically low frequency (3.4%) and occurred only in cultivar Meteor. Such limited representation of the PVY resistance gene highlights the importance of involving cultivar Meteor as a donor of this valuable allele in breeding programs. Population analysis of marker frequency revealed a high level of genetic resistance in the studied hybrid material to *S. endobioticum* and *G. rostochiensis*. The level of enrichment with the *H1 *(57R and N195) and *Sen1* (NL25) loci exceeded 70%. At the same time, the critical deficiency of PVY resistance genes indicates the need to adjust selection of parental pairs in future breeding cycles. The obtained frequency data may serve as a basis for estimating breeding genetic distances and predicting selection shifts in subsequent generations. Effective pyramiding of the *Rx1* gene can be achieved through direct selection, whereas breeding for the *Gpa2* locus requires more complex schemes involving progeny evaluation. The monomorphic state of the *Rysto* and *H1* (57R) loci highlights the need for substantial renewal of the parental pool. This may introduce polymorphism and restore effective selection for resistance to these pathogens. The obtained results enabled targeted selection of parental pairs for combinatory breeding. Hybridization between genotypes belonging to contrasting megaclusters may increase genetic diversity within the population. Crosses between such genotypes may facilitate effective pyramiding of markers associated with resistance to nematode species, *S. endobioticum*, and PVX, within a single progeny.

## Figures and Tables

**Table 1:** Arrangement of solutions and consumables in a 96-well deep-well plate for automated DNA purification using magnetic particles and the King Fisher Duo Prime processor.

A	Elution Solution (120 µL Per Well)
B	Magnetic rod tip comb
C	Sample: 500 µL lysate, 450 µL precipitation solution, 50 µL magnetic particles per well
D	Empty
E	Wash solution 1 (500 µL per well)
F	Wash solution 2 (500 µL per well)
G	Wash solution 3 (300 µL per well)
H	Empty

**Table 2:** Characteristics of DNA markers used for the identification of potato genes conferring resistance to pathogens and pests.

DNA Markers Associated with Potato Resistance to Pathogens				
Gene	Marker	Primer	Fragment	MgCl_2_ (mM)
DNA markers associated with potato resistance to *G. rostochiensis*				
*H1*	N195	TGGAAATGGCACCCACTA CATCATGGTTTCACTTGTCAC	337	0.8
	57 R (SCAR)	F: TGCCTGCCTCTCCGATTTCT R: GGTTCAGCAAAAGCAAGGACGTG	450	0.7
DNA markers associated with potato resistance to *G. pallida*				
*Gpa2*	Gpa2-2 (STS)	GCACTTAGAGACTCATTCCA ACAGATTGTTGGCAGCGAAA	452	2
DNA markers associated with potato resistance to *S. endobioticum*				
*Sen1*	NL 25 (SCAR)	TATTGTTAATCGTTACTCCCTC AGAGTCGTTTTACCGACTCC	1400	1
DNA markers associated with potato resistance to *PVX*				
*Rx1*	PVX (STS)	ATCTTGGTTTGAATACATGG CACAATATTGGAAGGATTCA	1230	2.5
DNA markers associated with potato resistance to *PVY*				
*Rysto*	YES3-3A (STS)	TAACTCAAGCGGAATAACCC AATTCACCTGTTTACATGCTTCTTGTG	341	0.8

## Data Availability

The original contributions presented in this study are included in the article. Further inquiries can be directed to the corresponding author.
